# Associations between the Dietary Inflammatory Index and Sleep Metrics in the Energy Balance Study (EBS)

**DOI:** 10.3390/nu15020419

**Published:** 2023-01-13

**Authors:** Emily T. Farrell, Michael D. Wirth, Alexander C. McLain, Thomas G. Hurley, Robin P. Shook, Gregory A. Hand, James R. Hébert, Steven N. Blair

**Affiliations:** 1 Department of Epidemiology and Biostatistics, Arnold School of Public Health, University of South Carolina, Columbia, SC 29208, USA; 2 College of Nursing, University of South Carolina, Columbia, SC 29208, USA; 3 Cancer Prevention and Control Program, Arnold School of Public Health, University of South Carolina, Columbia, SC 29208, USA; 4 Department of Pediatrics, Children’s Mercy, Kansas City, MO 64108, USA; 5 School of Medicine, University of Missouri-Kansas City, Kansas City, MO 64108, USA; 6 College of Health Professions, Wichita State University, Wichita, KS 67260, USA; 7 Department of Nutrition Connecting Health Innovations LLC, Columbia, SC 29208, USA; 8 Department of Exercise Science, Arnold School of Public Health, University of South Carolina, Columbia, SC 29208, USA

**Keywords:** Dietary Inflammatory Index (DII), diet quality, wake-after-sleep-onset, bedtime, waketime, inflammation

## Abstract

(1) Background: Sleep, a physiological necessity, has strong inflammatory underpinnings. Diet is a strong moderator of systemic inflammation. This study explored the associations between the Dietary Inflammatory Index (DII^®^) and sleep duration, timing, and quality from the Energy Balance Study (EBS). (2) Methods: The EBS (*n* = 427) prospectively explored energy intake, expenditure, and body composition. Sleep was measured using BodyMedia’s SenseWear^®^ armband. DII scores were calculated from three unannounced dietary recalls (baseline, 1-, 2-, and 3-years). The DII was analyzed continuously and categorically (very anti-, moderately anti-, neutral, and pro-inflammatory). Linear mixed-effects models estimated the DII score impact on sleep parameters. (3) Results: Compared with the very anti-inflammatory category, the pro-inflammatory category was more likely to be female (58% vs. 39%, *p* = 0.02) and African American (27% vs. 3%, *p* < 0.01). For every one-unit increase in the change in DII score (i.e., diets became more pro-inflammatory), wake-after-sleep-onset (WASO) increased (βChange = 1.00, *p* = 0.01), sleep efficiency decreased (βChange = −0.16, *p* < 0.05), and bedtime (βChange = 1.86, *p* = 0.04) and waketime became later (βChange = 1.90, *p* < 0.05). Associations between bedtime and the DII were stronger among African Americans (βChange = 6.05, *p* < 0.01) than European Americans (βChange = 0.52, *p* = 0.64). (4) Conclusions: Future studies should address worsening sleep quality from inflammatory diets, leading to negative health outcomes, and explore potential demographic differences.

## 1. Introduction

Despite the National Sleep Foundation’s recommendations stating adults ages 18 years or older should get more than seven hours of sleep per night [1], 35% of Americans do not meet the recommended sleep requirements [2]. Roughly 30–40% of Americans report insomnia symptoms during a given year [3]. Getting proper sleep (i.e., sufficient quality and duration, as well as timing that matches the body’s endogenous clock) is imperative as it is related to improvements in cerebral plasticity [4], memory, brain functioning, and physical restoration [5,6,7]. Later bedtime is associated with being overweight or obese, shorter sleep duration, and poorer eating behaviors in young adults [8,9]. Poor sleep quality and short sleep duration are associated with many chronic diseases and poor health outcomes, such as type 2 diabetes, coronary heart disease (CHD), cardiovascular disease (CVD), and obesity [10,11].

Current standard practice to treat sleep-related disorders or symptoms are pharmacological (e.g., benzodiazepines and non-benzodiazepine receptor agonists) [12]. The downside of such treatments is that they can be habit-forming and lead to a range of adverse side-effects. Such side effects can include increased incidence of falls and fractures, dementia and other memory/cognition issues, infections, and mortality [12]. More common side effects include excessive sleepiness and daytime fatigue, which are observed in more than 10% of those taking pharmacological treatments [13,14]. Non-pharmacological approaches to improve sleep are usually behavioral, such as ear plugs or eye masks to reduce sensory input, mindfulness, exercise, and cognitive behavioral therapy [15]. However, dietary improvements may be a prudent and effective approach, as studies on the association between sleep and diet show fairly clear and consistent associations. For example, obtaining recommended sleep duration (i.e., 7–8 h per night) is associated with greater nutrient intake [16,17]. It is known that high-fat diets are associated with several sleep disorders [16,17], and high-carbohydrate diets can decrease slow-wave sleep (SWS) and increase rapid-eye-movement (REM) sleep [16,18].

Sleep has strong inflammatory underpinnings. For example, both tumor necrosis factor (TNF)-α and interleukin (IL)-1β promote sleep [19,20,21,22,23]. IL-6 and soluble IL-6 receptor peak during REM sleep, while other cytokines peak during slow-wave sleep (SWS) [22,23]. In a meta-analysis of 72 studies, it was found that short sleep (i.e., <7 h) or long sleep (i.e., >8 h), compared with 7–8 h of sleep, was associated with elevated pro-inflammatory markers [24]. Additionally, a range of sleep disturbances are associated with increased inflammation [19,25,26]. Diet is one of the strongest moderators of chronic, systemic inflammation. Mediterranean diets (i.e., moderate-to-high intake of vegetables, fruits, nuts, unrefined grains, and fish) [27] are associated with lower inflammation. However, diets such as the Standard American Diet (SAD) tend to be more pro-inflammatory [28]. All in all, an overarching direct relationship between diet quality and sleep quality has become accepted [29].

The Dietary Inflammatory Index (DII^®^) was developed to quantify the inflammatory potential of various diets and to examine associations with a range of inflammation-related outcomes [30]. In previous studies utilizing the DII as a predictor for various aspects of sleep, a high DII score (i.e., indicating a pro-inflammatory diet) was associated with short sleep duration, self-reported sleep disturbances, high Pittsburgh Sleep Quality Index (PSQI) scores (i.e., “poor” sleep quality), higher wake-after-sleep-onset (WASO), obstructive sleep apnea severity (OSA), and daytime sleepiness and dysfunction [31,32,33,34,35].

The Energy Balance Study (EBS) was a comprehensive prospective cohort study designed to determine the association of caloric intake and energy expenditure on changes in body weight and composition in healthy adults [36]. A previous study based on the EBS found that greater body mass index (BMI) and body fat percentage were associated with poorer sleep efficiency and higher WASO, and those with greater BMI or body fat percentage also experience later wake times, shorter sleep duration, and longer sleep latency [37]. Given differences in energy intake, diet quality, and sleep duration and quality between races and sex, both race and sex were explored as potential effect modifiers [38,39,40,41,42,43,44,45,46,47,48]. The purpose of the current study is to evaluate whether associations exist between the DII and sleep metrics in the EBS and to test the hypothesis that more inflammatory diets would be associated with shorter sleep duration, worse sleep quality, and abnormal sleep timing. 

## 2. Materials and Methods

### 2.1. Study Population and Design

Data were extracted from the EBS (n at baseline = 427) over the course of up to three years of follow-up [36]. Clinical assessments, which included dietary recalls and actigraphy, occurred at baseline, then every three months for two years, and then every six months during the last year [36]. For the purposes of this analysis, data from baseline, one-year, two-year, and three-year timepoints were used. Participants lived in or near Columbia, South Carolina, were between 21 and 35 years old at baseline, and were normal weight (53.8%), overweight (31.5%), or obese (14.8%) BMIs (20–35 kg/m^2^) [36]. Those who planned to move out of the Columbia area; had major, acute, or chronic health conditions; or drastic weight changes prior to potential study participation were excluded [36]. The Energy Balance Study was approved by the institutional review board of the University of South Carolina, and all participants provided written informed consent.

### 2.2. Diet Ascertainment and the DII

Unannounced dietary 24-h recalls (24HR) were completed via telephone interviews, conducted by dietitians. During a two-week period, study participants were called on three randomly selected days. Recalls were to be obtained from two weekdays and one weekend day during each recall period. About 97% of participants completed at least two unannounced dietary recalls, contributing to the validity of the study’s DII estimates. The Nutrient Data System for Research (version 2012, Nutrition Coordinating Center, University of Minnesota, Minneapolis, MN, USA) was used to estimate average energy, nutrient, and individual food intakes from the 24HR. 

Development of the DII is described elsewhere [30]. However, DII score calculation for purposes of the present analyses is described hereafter. Of the possible 45 DII food parameters, 44 were used in this analysis; only eugenol was not available for use. Participants’ reported intakes of each of these DII food parameters (mostly micro and macronutrients with inclusion of some whole foods) was subtracted from a global mean from a composite database composed of dietary data from 11 populations around the world. Next, this difference was then divided by the global standard deviation, resulting in a z-score for each DII food parameter. The z-scores were converted to a proportion. They were then centered on 0 by doubling the proportion and subtracting 1. This centered proportion was then multiplied by article effect scores, which were derived from the review of nearly 2000 peer-reviewed research articles. These individual DII values for each food parameter indicate the degree of inflammatory potential for each parameter. Finally, these individual scores were summed to create the overall DII score. DII scores can theoretically range from −8 to +8. However, the range tends to be narrower in practice. More negative values are more anti-inflammatory and more positive numbers are more pro-inflammatory [49,50]. The E-DII was also calculated similarly but relied on an energy-adjusted global reference database. The difference between the E-DII and the DII is that the E-DII standardizes the global database and the participants’ intake per 1000 kcals consumed. Therefore, energy is not a separate DII food parameter in the E-DII, whereas it is for the DII. Depending on the population and eating behaviors within a population, either the DII or E-DII may be more predictive of study outcomes. Using Akaike information criterion values, it was found that use of the DII, compared with use of the E-DII, for this study, led to lower AIC values. Hence, only the DII was used in main analyses.

The DII score was treated both as continuous and categorical predictor in separate models (see statistical analysis for more details). DII was categorized as follows: DII 1 is “very anti-inflammatory” (<−3.0), DII 2 is “moderately anti-inflammatory” (−3.0 to −1.01), DII 3 is “neutral” (−1 to 0.99), and DII 4 is “pro-inflammatory” (>1.0). Typically, there would be a “very pro-inflammatory” category; however, the “pro-” and “very pro-” categories were combined due to the small number of participants with very pro-inflammatory diets. 

### 2.3. Sleep Assessment

Sleep was measured using BodyMedia’s SenseWear^®^ armband, which is a lightweight monitor that actively measures wear time, energy expenditure, steps, sleep, posture (indicating standing or laying down), and physical activity (duration, frequency, and intensity) [37,51]. These armbands use multiple sensors to detect such measurements: a heat flux sensor, galvanic skin response sensor, triaxial accelerometer, and thermistor-based skin surface sensor [37,51,52]. BodyMedia’s SenseWear^®^ armband is comparable to other actigraphy devices regarding sleep metrics, such as sleep duration, WASO, and sleep efficiency [37,53,54,55].

The definition of bedtime (i.e., sleep onset) was the first of three consecutive minutes spent asleep after lying down for at least 10 consecutive minutes. Sleep latency was the time between lying down and sleep onset. Wake-after-sleep-onset (WASO) was defined as the summation of waking minutes after sleep onset until sleep offset or waketime. It should be noted that to avoid periodic limb movement during sleep each waking period had to be at least two minutes in length to count towards WASO. Time in bed was the period between initial lying down time through to the final wake time. Nighttime sleep duration was the sum of all sleep-designated minutes during time in bed. Sleep offset or the final waketime was the first of 90 consecutive minutes spent awake. Sleep efficiency was then calculated as the nighttime sleep duration divided by the summation of sleep latency, sleep duration, and WASO. Social jetlag, which was also based on objective sleep measurements, was derived by subtracting the midpoint of sleep on the weekdays from the midpoint of sleep on the weekends.

The PSQI was used to determine subjective sleep medicine usage and sleep quality and quantity [56]. This self-administered survey contains 19 questions with seven component scores, and a global score. The global scores range from 0–21 with higher scores indicating worse sleep quality. A cut-point of 5.0, with values below 5 indicating good sleep quality, has been shown to have sensitivity of 90% and a specificity of 87% in identifying those with a sleep disorder. 

### 2.4. Covariates

Various potential covariates were assessed by utilizing subjective standardized assessments, such as the Perceived Stress Scale (PSS), Social Approval (SA), Social Desirability (SD) scores, and Eating Attitudes Questionnaire (EAQ). The PSS measures the degree to which an individual deems their life to be stressful [57]. SD measures self-reported socially desirable responses from an assessment of culturally approved behavior [58]. SA measures the need to obtain a positive response [59]. The EAQ measures eating disorder risk in three domains (1 = Cognitive Restraint, 2 = Disinhibition, 3 = Hunger) [60]. Additional covariates, such as BMI, waist-to-hip-ratio (WHR), diastolic and systolic blood pressure and blood composition, were assessed by standard research protocols [36]. All other potential covariates, such as gender, education, income, employment status, marital status, children, race, and age, were assessed from demographic questionnaires. Physical activity and sedentary hours were determined by objective measurements by wearing BodyMedia’s SenseWear Mini^®^ physical activity monitor, which were worn on average for 9.8 days (±0.9) for 23.2 ± 0.8 h/day [56].

### 2.5. Statistical Analysis

Analyses were conducted using SAS^®^ (version 9.4, Cary, NC, USA). Population characteristics were described using frequencies and percentages or means and standard deviations, as appropriate Chi-square tests and ANOVAs were used to compare population characteristics across DII categories. 

Two types of analytic procedures were conducted. The first was a mixed model with a random intercept that allowed both the dependent and independent variables to vary by time. These models estimated the association between the continuous form of the DII and sleep at any given point and are referred to as stationary effects models. The second approach compared the differential impacts of baseline DII (continuous) values and longitudinal DII changes on the sleep metrics. The longitudinal changes were defined as DII at later time points minus baseline (by definition, this is a continuous metric). For these models, the DII was treated as the independent variable and the dependent variables, fit in separate models, included WASO, sleep duration, sleep latency, sleep efficiency, bedtime, waketime, PSQI score, and social jetlag. Confounder selection started as a series of bivariate analyses (i.e., dependent = DII + covariate). The final model was achieved by a backward removal process. If removal of a variable led to a substantial (e.g., ±10% or more) change in the beta coefficient of the DII, the covariate was put back into the model; otherwise, it remained out. Statistically significant covariates also remained in the model. After examining model residuals, no violations of the assumptions of linear regression were found. The categorical DII was then used to obtain least square means of the dependent variables in the stationary effect models. For the longitudinal model, a contrast statement compared the baseline and longitudinal effects. Sex and race were then treated as potential effect modifiers. If the interaction between either of these measures and the DII was ≤0.20, results were further stratified to examine the relationship between the DII score and that specific sleep metric. Overall, statistically significant associations between DII scores, categorical or continuous, were described as *p* ≤ 0.05.

## 3. Results

### 3.1. General Participation Information

The study had a total number of 427 participants at baseline and a nearly equal number of males and females (49% and 51%, respectively). At baseline 84% were college educated, 48% had an income over $40,000, 44% were students, 46% were married, 85% had no children, and 13% were African American. The average age at baseline was 27.6 years (±3.8), the average BMI was 25.4 kg/m^2^ (±3.8), and the average WHR was 0.79 (±0.07). Compared with those in the Very Anti-inflammatory category, those in the Pro-inflammatory category were more likely to be females (58% vs. 39%, respectively, *p* = 0.02) and less likely to be European Americans (48% vs. 85%, respectively, *p* = <0.01). Those with more anti-inflammatory diets, compared to pro-inflammatory, were more likely to have lower PSS scores (*p* = 0.05), lower sedentary hours (*p* = 0.02), higher physical activity hours, and higher EAQ (Cognitive Restraint) scores (*p* < 0.01, Table 1).

### 3.2. Dietary Inflammatory Index and Stationary Effects

For the stationary effects models, it was found that, compared with those with a very anti-inflammatory diet, those with a pro-inflammatory diet had a later bedtime (anti-: 23:58 vs. pro-: 00:22 *p* < 0.01) and a later waketime (anti: 07:40 vs. pro-: 08:04; *p*< 0.01). No other differences were observed when the DII was used categorically with the other sleep metrics in the stationary effects model. For DII fit as a continuous variable, every one-unit increase in DII score was associated with an increase in wake after sleep onset (WASO) (β1: 0.797 (95%CI 0.11, 1.43); *p* = 0.02), bedtime (β1: 2.81 (95%CI 1.19, 4.49); *p* < 0.01), and wake time (β1: 2.85 (95%CI 1.20, 4.49); *p* < 0.01). In stationary effects models, a statistically significant interaction between DII score and race was noted for sleep duration (*p*= 0.04), bedtime (*p* = 0.16) and social jetlag (*p* = 0.05). When stratified by race, the relationship between the DII and sleep duration was not statistically significant among African Americans or European American. The association between DII score and bedtime was much (i.e., greater than three times) stronger among African Americans (β1= 5.94 (95%CI 1.22, 10.7); *p* = 0.01) compared with their European American counterparts (β1= 1.54 (95%CI −0.41, 3.49); *p* = 0.12). The same was true for social jetlag (African Americans: β1= 0.07 (95%CI 0.01, 0.14); *p* = 0.02, European Americans: β1= 0.02 (95%CI −0.01, 0.04); *p* = 0.34, data not tabulated). Stationary effects data are tabulated in Table 2.

### 3.3. Dietary Inflammatory Index and Longitudinal Effects

For longitudinal analyses, every one-unit increase in the change in DII score (i.e., becoming more pro-inflammatory over time), increased WASO (βChange= 1.00 (95%CI 0.26, 1.74); *p* = 0.01), decreased sleep efficiency (βChange= −0.16 (95%CI −0.32, −0.004); *p* = 0.05), incurred a later bedtime (βChange= 1.86 (95%CI 0.05, 3.68); *p* = 0.04), and incurred a later waketime (βChange= 1.90 (95%CI 0.04, 3.75); *p* = 0.04). Within longitudinal models, the baseline DII score was associated with later bedtime (βBase= 5.76 (95%CI 3.27, 8.26); *p* = <0.01) and a later waketime (βBase= 4.83 (95%CI 2.39, 7.26); *p* < 0.01). Interactions between the DII and race were found to be statistically significant for bedtime (*p* = 0.18), PSQI (*p* = 0.01), and social jetlag (*p* = 0.02). When stratified by race, the relationship between the change in DII and PSQI was not statistically significant in African Americans or European Americans (data not shown). The association between the change in the DII and bedtime was stronger among African Americans (βChange= 6.05 (95%CI 1.58, 10.12); *p* < 0.01) compared with their European American counterparts (βChange= 0.52 (95%CI −1.66, 2.69); *p* = 0.64. Findings suggest a positive association between the change in DII and social jetlag for African Americans (βChange= 0.080 (95%CI 0.003, 0.16); *p* = 0.04), but not for European Americans (βChange= 0.009 (95%CI −0.03, 0.04); *p* = 0.63, interaction results not tabulated). Longitudinal effect models are tabulated in Table 3.

## 4. Discussion

### 4.1. Main Findings

The EBS was designed to study energy intake and expenditure on body composition and examine the associations among caloric intake, energy expenditure, and other lifestyle metrics [36]. The current analyses from the EBS found that more pro-inflammatory diets were associated with greater WASO, decreased sleep efficiency, later bedtime and a later wake time. These generalizations of the results apply to both cross-sectional effects, as well as longitudinal changes over time with increasing pro-inflammatory DII scores. Additionally, the effect of the DII on bedtime was exacerbated or attenuated by race. African Americans were more likely to have stronger associations between the DII and bedtime (i.e., specifically later bedtimes with more pro-inflammatory diets) compared with their European American counterparts. Race was found to be a significant effect modifier between the DII and social jetlag, as African Americans’ change in DII (i.e., becoming more pro-inflammatory) had a greater impact on social jetlag (i.e., increasing social jetlag) compared with European Americans. 

### 4.2. Comparison with Other Studies

In stationary cross-sectional effects models, it is not possible to determine the direction of effect and it is likely that there is a bidirectional relationship between WASO and dietary inflammatory properties. It is possible that WASO affects diet quality. However, evidence is becoming increasingly clear that dietary quality, including micro and macronutrient composition, has a direct impact on sleep metrics [61,62]. The IMAGINE study, a longitudinal, dietary intervention among adults to reduce dietary inflammation, found significant associations between decreased dietary inflammatory properties and increased sleep onset latency, increased sleep efficiency, and decreased WASO, similar to the longitudinal analyses found in the present study [33]. Additionally, a study of women 40–60 years old found an association between increased WASO and increased inflammatory markers [63]. Although this population is older than the average participants in the EBS, results are comparable.

Similarly, evidence has shown an association between sleep timing (i.e., bedtime and waketime) and dietary quality [64,65]. Although not yet fully understood, the causal pathway between dietary quality and sleep timing is mediated by biological mechanisms and hormonal responses, including postprandial glucose [64,65]. Results from the PREDICT dietary intervention trial, composed of participants from the United Kingdom and the United States of America, found an association between increased postprandial glucose levels, which are described as a spike in the amount of glucose found in one’s plasma shortly after a meal that can contribute to cardiovascular and diabetic complications potentially causing an offset of circadian rhythms, and later sleep midpoint [65]. Additionally, a study assessing circadian systems and behavioral cycles (i.e., sleep/wake and fasting/feeding cycles) in healthy adults, including shift-workers, found that circadian misalignment, defined as a misalignment between the body’s internal circadian clock and 24-hour behavioral or environmental cycles (i.e., shift-workers, who sleep during the day and work and eat at night), increased postprandial glucose [66]. A dietary intervention study of healthy adult twins in Germany found that switching from a high-calorie, low-fat diet to a low-calorie, high-fat diet (i.e., more pro-inflammatory diet) negatively affected the functioning of the body’s circadian clock and expression of metabolic and inflammatory genes [67]. Finally, a 12-week dietary intervention in diabetic women utilizing the Dietary Approaches to Stop Hypertension (DASH) diet found that the intervention had a favorable impact on hormonal properties, including a decrease in postprandial glucose (2hPPG) [68]. Consistent with EBS analyses utilizing the DII, a decrease in dietary quality may have adverse effects on glucose levels and hormone responses, which may, in turn, cause a phase delay, as manifested in the current study as later bed and wake times.

The relationship between sleep efficiency and DII scores was found to be statistically significant, as an increase in DII score, or becoming more pro-inflammatory over time, was associated with a decline in sleep efficiency. For each one-unit increase in DII score (i.e., toward a more pro-inflammatory diet), sleep efficiency would decrease by 0.16%. The average sleep efficiency value across all DII categories ranges between 80.6 and 81.2%. Previous studies have indicated that diet and sleep efficiency have an inverse relationship [29,61,69,70]. An intervention study found that increased adherence to a higher quality diet, such as increased consumption of fruits and vegetables and adherence to a Mediterranean diet [71], showed an increase in sleep efficiency. Findings from this analysis of the EBS and DII indicate that the association between a pro-inflammatory diet and lower sleep efficiency is clinically significant and comparable to previous studies.

Race was used as an effect modifier between the DII and duration and timing of sleep. Although African Americans frequently report a greater affinity for morningness [72,73], the biological plausibility of the complex relationship between sleep timing including bedtime and race may be attributed to genetic influences that affect circadian rhythmic mechanisms and diet interactions that differ between African Americans and European Americans [38,39]. For example, African Americans have been found to typically have a higher blood pressure at night compared with European Americans [73]. This may serve as biological reasoning for African Americans typically having a later bedtime than their European American counterparts [74]. Additionally, African Americans, compared with their European American counterparts, have a disproportionate increase in their CLOCK gene expression due to poor dietary quality, thus disrupting circadian rhythms [38]. One randomized control trial assessing the impact of a Mediterranean diet, which are generally found to be anti-inflammatory [75], found that the maintenance of a Mediterranean diet had a protective effect against impacts of the CLOCK gene and increased hyperglycemia [39]. Similarly, African American’s social jetlag was impacted more by changes in DII score compared with their European American counterparts. Previous studies have found an association between race and social jetlag, as African Americans are more likely to have greater social jetlag than European Americans [76,77,78]. Additionally, associations between poor diet quality and social jetlag have been well established [79]. One study of university students from the UK, aged similar to the EBS population, found that those students who experienced social jetlag had poorer diet quality. Another study of adults aged 18–25 located in Barcelona, Spain found that those with lower adherence to a Mediterranean diet had higher social jetlag [80]. 

There is a growing body of literature indicating an association between dietary inflammatory potential and sleep. A cross-sectional study of Iranian female college students found an association between the odds of declining sleep quality (i.e., increased PSQI score) (OR = 1.22, 95%CI = 1.03, 1.44) as DII scores increased [35]. A study of southern Italian adults found that the odds of adequate sleep quality decreased as one’s diet became more pro-inflammatory (comparing DII category 1 to 4, OR = 0.49, 95% CI = 0.31, 0.78) [81]. A study analyzing the association between dietary inflammation and obstructive sleep apnea (OSA) severity among those diagnosed with OSA found that DII scores were predictive of OSA severity [32]. A cross-sectional study among United Arab Emirates college students found a direct association between E-DII score and daytime dysfunction [31]. Analyses of 2005–2016 National Health and Nutrition Examination Survey (NHANES) responses among adults found that those with the most pro-inflammatory diet (i.e., highest E-DII categorical score) had increased odds of ≤6 h of sleep (OR = 1.04, 95% CI = 1.21, 1.61), ≥9 h of sleep (OR = 1.23, 95% CI = 1.03, 1.46), and sleep disturbances (OR = 1.14, 95% CI = 1.02, 1.27) compared with those with the most anti-inflammatory diets (i.e., lowest E-DII score) [34].

Study populations differ due to setting, culture, and demographics compared with those in the EBS. Therefore, caution is warranted when comparing current EBS DII analyses to results from previous studies that utilized subjective sleep questionnaires, as objective sleep measurements in EBS are much more rigorous. However, there were three other E-DII studies that used similar sleep measurements for similar analyses. The aforementioned IMAGINE study found similar associations between DII score and sleep metrics noted in this study [33]. Despite the fact that the IMAGINE study was a clinical trial and had a different population demographic, results were strikingly similar, indicating consistency in such findings [33]. The Buffalo Cardio-Metabolic Occupational Police Stress (BCOPS) study studied longitudinal and cross-sectional E-DII scores and sleep metrics among police officers [82]. For longitudinal and cross-sectional effects, WASO increased as E-DII increased, indicating poorer sleep quality with worsening dietary inflammatory potential [82]. Although the BCOPS study design was similar to the EBS, the study population of police officers greatly differs from the young, healthy population studied in the EBS, as police officers are frequently exposed to stressors that may contribute to an increased likelihood of poor diet quality and poor sleep, such as shift-work, compared with the general population [82]. Lastly, the Health in Pregnancy and Postpartum (HIPP) study collected dietary, sleep and other demographic and lifestyle information among overweight or obese women during pregnancy [83]. Among those with a more pro-inflammatory diet, sleep latency was longer and, for European Americans, WASO was longer [83]. While the HIPP study population differs greatly from the EBS, as all study participants were pregnant women, associations between a pro-inflammatory diet and poor sleep persist, similar to the present study findings [83]. 

### 4.3. Strengths and Limitations

Strengths of the EBS study design include being able to establish temporality and changes in diet quality and sleep metrics across longitudinal analyses, large sample size, multi-year data collection, and data on multiple potential confounders. Unannounced 24-hour dietary recalls are considered the gold standard in dietary reporting. The subjective measure of sleep via the PSQI is from a peer-reviewed, validated questionnaire. BodyMedia’s SenseWear^®^ armband, which tracked objective sleep measures, physical activity, sedentary hours, and social jetlag, is comparable to other actigraphy devices and polysomnography [36]. Some limitations of the EBS were loss-to-follow-up and population generalizability. As the EBS consisted of primarily healthy, young adults who typically had no children and were unmarried, findings may not be generalizable to all other populations. Unannounced 24HR interviews are subject to some limitations, as self-reported diet can have inaccuracies due to the misremembering of food and drink or social desirability bias. The PSQI is subject to response bias, as participants may inaccurately report their sleep measures

## 5. Conclusions

Analyses from EBS found that increased WASO, decreased sleep efficiency, later bedtime, and later waketime were significantly associated with higher DII scores (i.e., indicating a more pro-inflammatory diet). Future work should focus on the question of whether worsening sleep quality due to poor dietary quality leads to further downstream health effects. 

## Figures and Tables

**Table 1:** Baseline population characteristics overall and by Dietary Inflammatory Index categories among all participants of the Energy Balance Study.

Frequency (%) or Mean ± St. Dev						
Characteristics	All Participants	DII–1	DII–2	DII–3	DII–4	*p*-Value *
**Gender**	427					**0** **.02**
Male	210 (49%)	60 (61%)	55 (52%)	45 (43%)	50 (42%)	
Female	217 (51%)	39 (39%)	51 (48%)	59 (57%)	68 (58%)	
**Education**	427					0.13
<3 years of college	70 (16%)	11 (11%)	19 (18%)	14 (13%)	26 (22%)	
4+ years of college	357 (84%)	88 (89%)	87 (82%)	90 (87%)	92 (78%)	
**Income ($USD)**	425					**<0.01**
0–19,999	71 (17%)	14 (14%)	18 (17%)	15 (14%)	24 (20%)	
20,000–39,999	148 (35%)	27 (28%)	27 (25%)	46 (44%)	48 (41%)	
40,000–59,999	85 (20%)	15 (15%)	29 (27%)	16 (15%)	25 (21%)	
60,000–80,000	53 (12%)	13 (13%)	16 (15%)	10 (10%)	14 (12%)	
80,000+	68 (16%)	28 (29%)	16 (15%)	17 (16%)	7 (6%)	
**Employment**	426					0.15
Non-student	237 (56%)	64 (65%)	60 (57%)	54 (52%)	59 (50%)	
Student	189 (44%)	35 (35%)	46 (43%)	49 (48%)	59 (50%)	
**Marital**	427					0.07
Married/living with someone	197 (46%)	56 (57%)	47 (44%)	48 (46%)	46 (39%)	
Single	230 (54%)	43 (43%)	59 (56%)	56 (54%)	72 (61%)	
**Children**	426					0.35
0	363 (85%)	89 (90%)	95 (90%)	83 (80%)	96 (82%)	
1	35 (8%)	6 (6%)	4 (4%)	12 (12%)	13 (11%)	
2	20 (5%)	2 (2%)	6 (6%)	7 (7%)	5 (4%)	
3	4 (1%)	0 (0%)	1 (1%)	1 (1%)	2 (2%)	
4	4 (1%)	2 (2%)	0 (0%)	1 (1%)	1 (1%)	
**Race**	427					**<0.01**
European-American	284 (67%)	84 (85%)	71 (67%)	72 (69%)	57 (48%)	
African American	54 (13%)	3 (3%)	13 (12%)	6 (6%)	32 (27%)	
Other	89 (21%)	12 (12%)	22 (21%)	26 (25%)	29 (26%)	
**Sleep Med Use ^1^**	427					0.41
Not during the past month	366 (86%)	87 (88%)	86 (81%)	92 (89%)	101 (86%)	
Less than once a week	61 (14%)	12 (12%)	20 (19%)	12 (12%)	17 (14%)	
**Age**		99	106	104	118	0.12
Mean ± STE	27.6 ± 3.8	28.3 ± 3.5	27.7 ± 3.9	27.0 ± 3.6	27.6 ± 4.0	
**BMI ^2^** (kg/m^2^)						0.22
Mean ± STE	25.4 ± 3.8	25.1 ± 3.5	25.0 ± 3.5	25.5 ± 4.0	25.9 ± 4.2	
**Systolic BP ^3^**						0.06
Mean ± STE	122.8 ± 11.9	125.0 ± 11.5	123.0 ± 12.2	123.1 ± 12.8	120.6 ± 10.7	
**Diastolic BP ^3^**						0.51
Mean ± STE	73.7 ± 8.8	74.4 ± 8.7	74.1 ± 8.6	73.6 ± 9.8	72.7 ± 8.2	
**WHR ^4^**						0.58
Mean ± STE	0.79 ± 0.070	0.80 ± 0.072	0.79 ± 0.07	0.79 ± 0.07	0.79 ± 0.07	
**PSS ^5^ Score**						**0** **.05**
Mean ± STE	12.5 ± 5.5	11.2 ± 5.4	12.8 ± 5.9	12.7 ± 5.0	13.1 ± 5.6	
**SA ^6^ Score**						0.93
Mean ± STE	51.5 ± 9.3	51.4 ± 9.5	51.2 ± 9.4	51.3 ± 8.2	51.9 ± 10.0	
**SD ^7^ Score**						0.09
Mean ± STE	18.5 ± 5.2	17.7 ± 5.0	19.3 ± 5.0	18.1 ± 5.2	19.0 ± 5.4	
**Sedentary Hours**						**0** **.02**
Mean ± STE	18.1 ± 1.5	17.9 ± 1.5	18.1 ± 1.4	18.0 ± 1.5	18.5 ± 1.3	
**Physical** **Activity Hours**						**0** **.01**
Mean ± STE	2.3 ± 1.3	2.5 ± 1.4	2.4 ± 1.2	2.3 ± 1.4	1.9 ± 1.1	
**Social Jet Lag**						0.97
Mean ± STE	1.2 ± 0.8	1.1 ± 0.7	1.2 ± 0.8	1.2 ± 0.9	1.2 ± 0.9	
**EAQ ^8^ 1**						**<0.01**
Mean ± STE	10.3 ± 4.9	11.3 ± 4.7	10.7 ± 4.8	10.2 ± 4.9	9.0 ± 4.9	
**EAQ ^8^ 2**						0.87
Mean ± STE	5.1 ± 2.9	5.0 ± 3.0	5.0 ±2.8	5.2 ± 2.7	5.3 ± 3.1	
**EAQ ^8^ 3**						0.31
Mean ± STE	5.07 ± 3.2	5.3 ± 3.3	5.0 ± 3.3	5.4 ± 3.2	4.7 ± 2.9	

* Bold *p*-values indicate statistically significant values (*p* < 0.05). ^1^ Sleep med use was measured by the Pittsburgh Sleep Quality Index (PSQI);^2^ BMI = body mass index; ^3^ BP = blood pressure; ^4^ WHR = waist-to-hip ratio; ^5^ PSS = Perceived Stress scale; ^6^ SA = social approval; ^7^ SD = social desirability; ^8^ EAQ = Eating Attitudes Questionnaire.

**Table 2:** Sleep metrics and dietary inflammatory indices per participant.

Mean (Minimum–Maximum) or (95% CL)							
Sleep Metric	DII Category						
	Very Anti- Inflammatory	Moderately Anti- Inflammatory	Neutral	Pro- Inflammatory	*p*-Value: * Very Anti vs. Pro	DII Continuous Beta	*p*-Value: * Continuous
**WASO ^1^** (min)	57.9 (53.6–62.2)	61.2 (57.2–65.2)	62.3 (58.3–57.7)	61.9 (57.7–66.2)	0.75	0.7966 (0.11–1.43)	**0** **.02**
**Sleep duration** (hours)	6.45 (6.32–6.59)	6.43 (6.31–393.34)	6.40 (6.27–6.53)	6.34 (6.21–6.47)	0.12	−0.8567 (−2.04–0.33)	0.16
**Sleep latency** (min)	12.9 (12.1–13.7)	13.1 (12.4–13.9)	13.0 (12.2–13.8)	13.2 (12.3–14.1)	0.70	0.005866 (−0.15–0.16)	0.94
**Sleep** **efficiency** (%)	81.2 (80.2–82.2)	80.7 (79.7–81.6)	80.6 (79.6–81.5)	80.6 (79.6–81.6)	0.23	−0.1247 (−0.27–0.02)	0.09
**Bedtime** (00:00–23:59)	23:58 (23:45–00:11)	00:07 (23:54–00:18)	00:11 (23:59–00:22)	00:22 (00:11–00:34)	**<0** **.01**	2.8103 (1.19–4.44)	**<0** **.01**
**Waketime** (00:00–23:59)	07:40 (07:27–07:52)	07:50 (07:38–08:01)	07:49 (07:39–08:01)	08:04 (07:53–08:16)	**<0** **.01**	2.8459 (1.20–4.49)	**<0** **.01**
**PSQI ^2^ Sleep score**	5.11 (4.80–5.43)	5.21 (4.91–5.51)	5.45 (5.14–5.76)	5.36 (5.02–5.70)	0.23	0.04967 (−0.01–0.10)	0.08
**Social jetlag**	1.11 (0.97–1.24)	1.25 (1.12–1.38)	1.37 (1.00–1.27)	1.25 (1.10–1.40)	0.07	0.01016 (−0.01–0.03)	0.34

* Bold *p*-values indicate statistically significant values (*p* < 0.05). ^1^ WASO = wake after sleep onset; ^2^ PSQI = Pittsburgh Sleep Quality Index.

**Table 3:** Longitudinal changes and baseline effects of sleep metrics from dietary inflammatory indices.

Sleep Metric	β_Change_ (95% CL)	*p*-Value * β_Change_	β_Base_ (95% CL)	*p*-Value * β_Base_	*p*-Value * β_Change_ vs. β_Base_
**WASO ^1^** (min)	1.0002 (0.26–1.74)	**0** **.01**	0.1958 (−0.84–1.23)	0.71	0.16
**Sleep duration** (hours)	−0.7796 (−2.15–0.59)	0.26	−0.8114 (−2.56–0.94)	0.36	0.97
**Sleep latency** (min)	−0.01907 (−0.21–0.17)	0.85	−0.00317 (−0.20–0.20)	0.96	0.89
**Sleep efficiency** (%)	−0.1626 (−0.32–−0.004)	**0** **.05**	−0.05328 (−0.29–0.17)	0.66	0.41
**Bedtime** (00:00–23:59)	1.8647 (0.05–3.68)	**0.04**	5.7605 (3.27–8.26)	**<0.01**	**<0.01**
**Waketime** (00:00–23:59)	1.8958 (0.04–3.75)	**0.05**	4.8252 (2.39–7.26)	**<0.01**	**0.03**
**PSQI ^2^ Sleep score**	0.04163 (−0.03–0.11)	0.23	0.06511 (−0.004–0.13)	0.07	0.57
**Social jetlag**	0.001611 (−0.03–0.03)	0.91	0.01814 (−0.005–0.04)	0.12	0.26

* Bold *p*-values indicate statistically significant values (*p* < 0.05). ^1^ WASO = wake after sleep onset; ^2^ PSQI = Pittsburgh Sleep Quality Index.

## Data Availability

The Energy Balance study data are available upon request. The Energy Balance study principal investigators should be contacted for data availability.

## References

[1] Hirshkowitz M., Whiton K., Albert S.M., Alessi C., Bruni O., DonCarlos L., Hazen N., Herman J., Katz E.S., Kheirandish-Gozal L. (2015). National sleep foundation’s sleep time duration recommendations: Methodology and results summary. Sleep Health.

[2] National Center for Chronic Disease Prevention and Health Promotion Short Sleep Duration Among US Adults. https://www.cdc.gov/sleep/data_statistics.html.

[3] Dopheide J.A. (2020). Insomnia overview: Epidemiology, pathophysiology, diagnosis and monitoring, and nonpharmacologic therapy. Am. J. Manag. Care.

[4] Dang-Vu T.T., Desseilles M., Peigneux P., Maquet P. (2006). A role for sleep in brain plasticity. Pediatr. Rehabil..

[5] Cai D.J., Rickard T.C. (2009). Reconsidering the role of sleep for motor memory. Behav. Neurosci..

[6] Walker M.P. (2008). Cognitive consequences of sleep and sleep loss. Sleep Med..

[7] Driver H.S., Taylor S.R. (2000). Exercise and sleep. Sleep Med. Rev..

[8] Grummon A.H., Sokol R.L., Lytle L.A. (2021). Is late bedtime an overlooked sleep behaviour? Investigating associations between sleep timing, sleep duration and eating behaviours in adolescence and adulthood. Public Health Nutr..

[9] Shochat T., Cohen-Zion M., Tzischinsky O. (2014). Functional consequences of inadequate sleep in adolescents: A systematic review. Sleep Med. Rev..

[10] Lu C., Liao B., Nie J., Wang W., Wang Y. (2020). The association between sleep duration and chronic diseases: A population-based cross-sectional study. Sleep Med..

[11] Jike M., Itani O., Watanabe N., Buysse D.J., Kaneita Y. (2018). Long sleep duration and health outcomes: A systematic review, meta-analysis and meta-regression. Sleep Med. Rev..

[12] Brandt J., Leong C. (2017). Benzodiazepines and Z-Drugs: An Updated Review of Major Adverse Outcomes Reported on in Epidemiologic Research. Drugs RD.

[13] Holbrook A.M., Crowther R., Lotter A., Cheng C., King D. (2000). Meta-analysis of benzodiazepine use in the treatment of insomnia. Can. Med. Assoc. J..

[14] Proctor A., Bianchi M.T. (2012). Clinical Pharmacology in Sleep Medicine. ISRN Pharmacol..

[15] Miller M.A., Renn B.N., Chu F., Torrence N. (2019). Sleepless in the hospital: A systematic review of non-pharmacological sleep interventions. Gen. Hosp. Psychiatry.

[16] St-Onge M.-P., Mikic A., Pietrolungo C.E. (2016). Effects of Diet on Sleep Quality. Adv. Nutr. Int. Rev. J..

[17] Grandner M.A., Jackson N., Gerstner J.R., Knutson K.L. (2013). Dietary nutrients associated with short and long sleep duration. Data from a nationally representative sample. Appetite.

[18] Afaghi A., O’Connor H., Chow C.M. (2007). High-glycemic-index carbohydrate meals shorten sleep onset. Am. J. Clin. Nutr..

[19] Kapsimalis F., Basta M., Varouchakis G., Gourgoulianis K., Vgontzas A., Kryger M. (2008). Cytokines and pathological sleep. Sleep Med..

[20] Krueger J.M. (2003). Biochemical regulation of non-rapid-eye-movement sleep. Front. Biosci..

[21] Krueger J.M., Majde J.A. (2003). Humoral Links between Sleep and the Immune System. Ann. New York Acad. Sci..

[22] Irwin M.R. (2019). Sleep and inflammation: Partners in sickness and in health. Nat. Rev. Immunol..

[23] Redwine L., Hauger R.L., Gillin J.C., Irwin M. (2000). Effects of Sleep and Sleep Deprivation on Interleukin-6, Growth Hormone, Cortisol, and Melatonin Levels in Humans^1^. J. Clin. Endocrinol. Metab..

[24] Irwin M.R., Olmstead R., Carroll J.E. (2016). Sleep Disturbance, Sleep Duration, and Inflammation: A Systematic Review and Meta-Analysis of Cohort Studies and Experimental Sleep Deprivation. Biol. Psychiatry.

[25] Kheirandish-Gozal L., Gozal D. (2019). Obstructive Sleep Apnea and Inflammation: Proof of Concept Based on Two Illustrative Cytokines. Int. J. Mol. Sci..

[26] Slavish D.C., Graham-Engeland J.E., Engeland C.G., Taylor D.J., Buxton O.M. (2018). Insomnia symptoms are associated with elevated C-reactive protein in young adults. Psychol. Health.

[27] Trichopoulou A., Costacou T., Bamia C., Trichopoulos D. (2003). Adherence to a Mediterranean Diet and Survival in a Greek Population. N. Engl. J. Med..

[28] Ahluwalia N., Andreeva V., Kesse-Guyot E., Hercberg S. (2013). Dietary patterns, inflammation and the metabolic syndrome. Diabetes Metab..

[29] Vernia F., Di Ruscio M., Ciccone A., Viscido A., Frieri G., Stefanelli G., Latella G. (2021). Sleep disorders related to nutrition and digestive diseases: A neglected clinical condition. Int. J. Med. Sci..

[30] Shivappa N., Steck S.E., Hurley T.G., Hussey J.R., Ma Y., Ockene I.S., Tabung F., Hebert J.R. (2014). A population-based dietary inflammatory index predicts levels of C-reactive protein in the Seasonal Variation of Blood Cholesterol Study (SEASONS). Public Health Nutr..

[31] Masaad A.A., Yusuf A.M., Shakir A.Z., Khan M.S., Khaleel S., Ismail L.C., Faris M.A.-I.E., Jahrami H.A., Shivappa N., Hebert J.R. (2021). Sleep quality and Dietary Inflammatory Index among university students: A cross-sectional study. Sleep Breath..

[32] Lopes T.V., Borba M.E., Lopes R.V., Fisberg R.M., Paim S.L., Teodoro V.V., Zimberg I.Z., Araújo L.B., Shivappa N., Hébert J.R. (2019). Association between inflammatory potential of the diet and sleep parameters in sleep apnea patients. Nutrition.

[33] Wirth M.D., Jessup A., Turner-McGrievy G., Shivappa N., Hurley T.G., Hébert J.R. (2020). Changes in dietary inflammatory potential predict changes in sleep quality metrics, but not sleep duration. Sleep.

[34] Kase B.E., Liu J., Wirth M.D., Shivappa N., Hebert J.R. (2021). Associations between dietary inflammatory index and sleep problems among adults in the United States, NHANES 2005–2016. Sleep Health.

[35] Bazyar H., Javid A.Z., Behbahani H.B., Shivappa N., Hebert J.R., Khodaramhpour S., Zadeh S.K., Aghamohammadi V. (2021). The association between dietary inflammatory index with sleep quality and obesity amongst iranian female students: A cross-sectional study. Int. J. Clin. Pract..

[36] Hand G.A., Shook R.P., Paluch A.E., Baruth M., Crowley E.P., Jaggers J.R., Prasad V.K., Hurley T.G., Hebert J.R., O’Connor D.P. (2013). The Energy Balance Study: The Design and Baseline Results for a Longitudinal Study of Energy Balance. Res. Q. Exerc. Sport.

[37] Wirth M.D., Hébert J.R., Hand G.A., Youngstedt S.D., Hurley T.G., Shook R.P., Paluch A.E., Sui X., James S.L., Blair S.N. (2015). Association between actigraphic sleep metrics and body composition. Ann. Epidemiol..

[38] Riestra P., Gebreab S.Y., Xu R., Khan R.J., Gaye A., Correa A., Min N., Sims M., Davis S.K. (2017). Circadian CLOCK gene polymorphisms in relation to sleep patterns and obesity in African Americans: Findings from the Jackson heart study. BMC Genet..

[39] Corella D., Asensio E.M., Coltell O., Sorlí J.V., Estruch R., Martínez-González M., Salas-Salvadó J., Castañer O., Arós F., Lapetra J. (2016). CLOCK gene variation is associated with incidence of type-2 diabetes and cardiovascular diseases in type-2 diabetic subjects: Dietary modulation in the PREDIMED randomized trial. Cardiovasc. Diabetol..

[40] Reyner A., Horne J.A. (1995). Gender- and Age-Related Differences in Sleep Determined by Home-Recorded Sleep Logs and Actimetry From 400 Adults. Sleep.

[41] Burgard S.A., Ailshire J.A. (2013). Gender and Time for Sleep among U.S. Adults. Am. Sociol. Rev..

[42] Tonetti L., Fabbri M., Natale V. (2008). Sex Difference in Sleep-Time Preference and Sleep Need: A Cross-Sectional Survey among Italian Pre-Adolescents, Adolescents, and Adults. Chrono. Int..

[43] Glavin E.E., Matthew J., Spaeth A.M. (2022). Gender Differences in the Relationship Between Exercise, Sleep, and Mood in Young Adults. Health Educ. Behav..

[44] Duffy J.F., Cain S.W., Chang A.-M., Phillips A.J.K., Münch M.Y., Gronfier C., Wyatt J.K., Dijk D.-J., Wright K.P., Czeisler C.A. (2011). Sex difference in the near-24-hour intrinsic period of the human circadian timing system. Proc. Natl. Acad. Sci. USA.

[45] Kang M., Park S.-Y., Shvetsov Y.B., Wilkens L.R., Le Marchand L., Boushey C.J., Paik H.-Y. (2019). Sex differences in sociodemographic and lifestyle factors associated with diet quality in a multiethnic population. Nutrition.

[46] McNaughton S.A., Ball K., Crawford D., Mishra G.D. (2008). An Index of Diet and Eating Patterns Is a Valid Measure of Diet Quality in an Australian Population. J. Nutr..

[47] Frazier-Wood A.C., Kim J., Davis J.S., Jung S.Y., Chang S. (2015). In cross-sectional observations, dietary quality is not associated with CVD risk in women; in men the positive association is accounted for by BMI. Br. J. Nutr..

[48] Wang D.D., Leung C.W., Li Y., Ding E., Chiuve S., Hu F.B., Willett W.C. (2014). Trends in Dietary Quality Among Adults in the United States, 1999 Through 2010. JAMA Intern. Med..

[49] Hébert J.R., Shivappa N., Wirth M.D., Hussey J.R., Hurley T.G. (2019). Perspective: The Dietary Inflammatory Index (DII)—Lessons Learned, Improvements Made, and Future Directions. Adv. Nutr. Int. Rev. J..

[50] Shivappa N., Steck S.E., Hurley T.G., Hussey J.R., Hébert J.R. (2014). Designing and developing a literature-derived, population-based dietary inflammatory index. Public Health Nutr..

[51] St-Onge M., Mignault D., Allison D., Rabasa-Lhoret R. (2007). Evaluation of a portable device to measure daily energy expenditure in free-living adults. Am. J. Clin. Nutr..

[52] Mignault D., St.-Onge M., Karelis A.D., Allison D.B., Rabasa-Lhoret R. (2005). Evaluation of the Portable HealthWear Armband: A device to measure total daily energy expenditure in free-living type 2 diabetic individuals. Diabetes Care.

[53] O’Driscoll D.M., Turton A.R., Copland J.M., Strauss B.J., Hamilton G. (2013). Energy expenditure in obstructive sleep apnea: Validation of a multiple physiological sensor for determination of sleep and wake. Sleep Breath..

[54] Bahammam A.S., Sharif M.M. (2013). Sleep estimation using BodyMedia′s SenseWear™ armband in patients with obstructive sleep apnea. Ann. Thorac. Med..

[55] Soric M., Turkalj M., Kucic D., Marusic I., Plavec D., Misigoj-Durakovic M. (2013). Validation of a multi-sensor activity monitor for assessing sleep in children and adolescents. Sleep Med..

[56] Wirth M.D., Hébert J.R., Shivappa N., Hand G.A., Hurley T.G., Drenowatz C., McMahon D., Shook R.P., Blair S.N. (2016). Anti-inflammatory Dietary Inflammatory Index scores are associated with healthier scores on other dietary indices. Nutr. Res..

[57] Cohen S., Kamarck T., Mermelstein R. (1983). A global measure of perceived stress. J. Health Soc. Behav..

[58] Hebert J.R., Clemow L., Pbert L., Ockene I.S., Ockene J.K. (1995). Social Desirability Bias in Dietary Self-Report May Compromise the Validity of Dietary Intake Measures. Leuk. Res..

[59] Adams S.A., Matthews C.E., Ebbeling C.B., Moore C.G., Cunningham J.E., Fulton J., Hebert J.R. (2005). The Effect of Social Desirability and Social Approval on Self-Reports of Physical Activity. Am. J. Epidemiol..

[60] Garner D.M., Olmsted M.P., Bohr Y., Garfinkel P.E. (1982). The Eating Attitudes Test: Psychometric features and clinical correlates. Psychol. Med..

[61] Wilson K., St-Onge M.-P., Tasali E. (2022). Diet Composition and Objectively Assessed Sleep Quality: A Narrative Review. J. Acad. Nutr. Diet..

[62] Peuhkuri K., Sihvola N., Korpela R. (2012). Diet promotes sleep duration and quality. Nutr. Res..

[63] Nowakowski S., Matthews K.A., von Känel R., Hall M.H., Thurston R.C. (2018). Sleep characteristics and inflammatory biomarkers among midlife women. Sleep.

[64] Chaput J.-P. (2014). Sleep patterns, diet quality and energy balance. Physiol. Behav..

[65] Tsereteli N., Vallat R., Fernandez-Tajes J., Delahanty L.M., Ordovas J.M., Drew D.A., Valdes A.M., Segata N., Chan A.T., Wolf J. (2022). Impact of insufficient sleep on dysregulated blood glucose control under standardised meal conditions. Diabetologia.

[66] Morris C.J., Yang J.N., Garcia J.I., Myers S., Bozzi I., Wang W., Buxton O.M., Shea S.A., Scheer F.A.J.L. (2015). Endogenous circadian system and circadian misalignment impact glucose tolerance via separate mechanisms in humans. Proc. Natl. Acad. Sci. USA.

[67] Pivovarova O., Jürchott K., Rudovich N., Hornemann S., Ye L., Möckel S., Murahovschi V., Kessler K., Seltmann A.-C., Maser-Gluth C. (2015). Changes of Dietary Fat and Carbohydrate Content Alter Central and Peripheral Clock in Humans. J. Clin. Endocrinol. Metab..

[68] Daneshzad E., Heshmati J., Basirat V., Keshavarz S.-A., Qorbani M., Larijani B., Bellissimo N., Azadbakht L. (2022). The Effect of the Dietary Approaches to Stop Hypertension (DASH) Diet on Sleep, Mental Health, and Hormonal Changes: A Randomized Clinical Trial in Women With Type 2 Diabetes. Front. Nutr..

[69] Tan X., Alén M., Wang K., Tenhunen J., Wiklund P., Partinen M., Cheng S. (2016). Effect of Six-Month Diet Intervention on Sleep among Overweight and Obese Men with Chronic Insomnia Symptoms: A Randomized Controlled Trial. Nutrients.

[70] Heath G., Dorrian J., Coates A. (2019). Associations between shift type, sleep, mood, and diet in a group of shift working nurses. Scand. J. Work. Environ. Health.

[71] Zuraikat F., Makarem N., St-Onge M.-P., Xi H., Akkapeddi A., Aggarwal B. (2020). A Mediterranean Dietary Pattern Predicts Better Sleep Quality in US Women from the American Heart Association Go Red for Women Strategically Focused Research Network. Nutrients.

[72] Randler C., Engelke J. (2019). Gender differences in chronotype diminish with age: A meta-analysis based on morningness/chronotype questionnaires. Chronobiol. Int..

[73] Malone S.K., Patterson F., Lozano A., Hanlon A. (2017). Differences in morning–evening type and sleep duration between Black and White adults: Results from a propensity-matched UK Biobank sample. Chronobiol. Int..

[74] Chung J., Goodman M., Huang T., Wallace M.L., Johnson D.A., Bertisch S., Redline S. (2021). Racial-ethnic Differences in Actigraphy, Questionnaire, and Polysomnography Indicators of Healthy Sleep: The Multi-Ethnic Study of Atherosclerosis. Am. J. Epidemiol..

[75] Mazidi M., Shivappa N., Wirth M.D., Hebert J.R., Mikhailidis D.P., Kengne A.P., Banach M. (2018). Dietary inflammatory index and cardiometabolic risk in US adults. Atherosclerosis.

[76] Malone S.K., Zemel B.S., Compher C., Souders M.C., Chittams J., Thompson A.L., Lipman T.H. (2016). Characteristics Associated With Sleep Duration, Chronotype, and Social Jet Lag in Adolescents. J. Sch. Nurs..

[77] Hayes J.F., Schumacher L.M., Lanoye A., LaRose J.G., Tate D.F., Espeland M.A., Gorin A.A., Lewis C.E., Jelalian E., Wing R.R. (2022). Persistent, High Levels of Social Jetlag Predict Poor Weight Outcomes in a Weight Gain Prevention Study for Young adults. J. Behav. Med..

[78] McMahon D.M., Burch J.B., Wirth M.D., Youngstedt S.D., Hardin J.W., Hurley T.G., Blair S.N., Hand G.A., Shook R.P., Drenowatz C. (2018). Persistence of social jetlag and sleep disruption in healthy young adults. Chronol. Int..

[79] Hall W.L. (2022). The emerging importance of tackling sleep–diet interactions in lifestyle interventions for weight management. Br. J. Nutr..

[80] Zerón-Rugerio M.F., Cambras T., Izquierdo-Pulido M. (2019). Social Jet Lag Associates Negatively with the Adherence to the Mediterranean Diet and Body Mass Index among Young Adults. Nutrients.

[81] Godos J., Ferri R., Caraci F., Cosentino F.I.I., Castellano S., Shivappa N., Hebert J.R., Galvano F., Grosso G. (2019). Dietary Inflammatory Index and Sleep Quality in Southern Italian Adults. Nutrients.

[82] Wirth M.D., Fekedulegn D., Andrew M.E., McLain A.C., Burch J.B., Davis J.E., Hébert J.R., Violanti J.M. (2021). Longitudinal and cross-sectional associations between the dietary inflammatory index and objectively and subjectively measured sleep among police officers. J. Sleep Res..

[83] Wirth M.D., Liu J., Wallace M.K.K., McLain A.C., Turner-McGrievy G.M., Davis J.E., Ryan N., Hébert J.R. (2022). Dietary Inflammatory Index and sleep quality and duration among pregnant women with overweight or obesity. Sleep.

